# Effects of feeding agroindustrial byproducts on rumen fermentation and microbiome of sheep

**DOI:** 10.3168/jdsc.2025-0914

**Published:** 2026-01-16

**Authors:** Carlos Navarro Marcos, Trinidad de Evan, Óscar Gonzalez Recio, Mónica Gutiérrez Rivas, María Dolores Carro

**Affiliations:** 1 Departamento de Producción Animal, Facultad de Veterinaria, Universidad Complutense de Madrid, Madrid, 28040, Spain; 2 Departamento de Producción Agraria, ETSIAAB, Universidad Politécnica de Madrid, Madrid, 28040, Spain; 3 Departamento de Mejora Genética Animal, Instituto Nacional de Investigación y Tecnología Agraria y Alimentaria–CSIC, Madrid, 28040, Spain

## Abstract

The use of agroindustrial byproducts in ruminant nutrition is gaining increased attention because of economic and environmental benefits and their potential to enhance ruminal function and animal performance. However, the effect of these byproducts on ruminal microbiome and fermentation has been scarcely investigated. This study aimed to evaluate the effects of replacing conventional feeds with agroindustrial byproducts (18:18:8 mixture of corn dried distillers grains with solubles, dry citrus pulp, and exhausted olive cake) on the ruminal microbiome and fermentation parameters of sheep. Four rumen-fistulated Lacaune sheep were used in 2 experimental periods, receiving mixed diets composed of 50% alfalfa hay and 50% of either a control concentrate (CON diet) or a concentrate with agroindustrial byproducts (BYP diet). Ruminal samples were collected at 0, 3, and 6 h postfeeding to assess microbial composition and fermentation parameters. Diet significantly influenced the ruminal microbiome, but the effect of individual sheep was more pronounced than that of the diet. Sampling time also influenced the ruminal microbiome. The inclusion of agroindustrial byproducts in the concentrate did not significantly alter ruminal fermentation parameters. However, the BYP diet led to more steady ruminal fermentation, preventing large fluctuations in total VFA and ammonia concentrations, likely the result of a broader range of fermentable substrates present in the BYP concentrate and changes in ruminal microbiome. These results may indicate improved synchronization of nutrient availability for ruminal microorganisms. Overall, replacing conventional feed ingredients with agroindustrial byproducts promoted microbial diversity and improved ruminal fermentation, potentially leading to increased dairy ruminant performance.

## Highlights

• Agroindustrial byproducts can reduce the environmental impact of farming systems.
• Feeding agroindustrial byproducts modifies the ruminal microbiome in sheep.
• Sheep fed byproducts show steadier VFA and ammonia concentrations postfeeding.
• Byproducts can improve nutrient synchronization and use by ruminal microorganisms.

## Graphical Abstract

**Figure:**
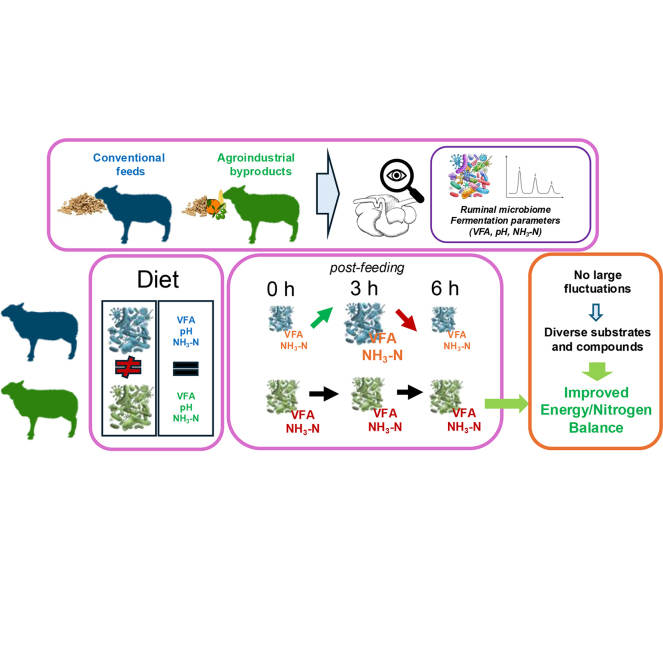
**Summary:** Dairy ruminants can contribute to the reduction of the negative environmental impact of the food industry by including agroindustrial byproducts (ABY) in their diets. We assessed the effects of replacing conventional feeds with ABY on the ruminal fermentation and ruminal microbiome of sheep. Feeding ABY significantly influenced the ruminal microbiome without altering fermentation parameters. Steadier volatile fatty acid (VFA) and ammonia concentrations were observed in ABY-fed sheep, suggesting improved nutrient synchronization and utilization by ruminal microorganisms. The use of ABY could help to reduce the environmental impact of both dairy ruminants and the food industry and improve nutrient utilization.

The use of agroindustrial byproducts (**ABY**) in ruminant animals is increasing globally because of cost reduction potential, noncompetition with human food, environmental benefits, and positive effects on animal health and product quality (Romero-Huelva et al., 2017; Marcos et al., 2020). Use of ABY in diets for ruminants is of special interest because they are often fibrous feeds that are more suitable for ruminants than for monogastric animals. Moreover, ABY frequently contain bioactive compounds that can modulate ruminal function, increasing nutrient digestibility, reducing acidosis incidence and methane production, and modulating fatty acid biohydrogenation, leading to beneficial changes in milk quality (Bryszak et al., 2019). In fact, multiple studies have demonstrated the positive effects of including ABY in diets for dairy ruminants (Karlsson et al., 2018; Carta et al., 2025). In a previous study (Marcos et al., 2020), replacing conventional feeds in the concentrate fed to dairy goats with a mixture of corn dried distiller grains with solubles, dry citrus pulp, and exhausted olive cake resulted in significant increases in the daily production of milk and milk concentrations of CP, fat, whey protein, TS and PUFA. However, the effects of including ABY in the diet of dairy ruminants are variable (Castellani et al., 2017; Romero-Huelva et al., 2017), which may be attributed to ABY heterogeneity, differences in ABY inclusion levels, or even to the animal species used in the different studies. Moreover, the bioactive compounds present in ABY can influence ruminal function, potentially leading to variable productive responses. Therefore, a better understanding of how ABY affect ruminal fermentation and the ruminal microbiome during the postprandial period could help to explain the observed effects. Therefore, the objective of this study was to evaluate the effect of replacing conventional ingredients in a concentrate for sheep with a mixture of ABY on ruminal microbiome and fermentation at different postprandial times.

All experiments were conducted in accordance with the Spanish regulations for experimental animal protection (approval number PROEX 035/17). Four rumen-fistulated Lacaune sheep (64.3 ± 2.11 kg of BW) were used in a crossover design with 2 experimental periods and 2 mixed diets. Sheep were housed individually in floor pens with continuous access to fresh water and a mineral and vitamin block over the trial. The 2 experimental diets consisted of a 50:50 mixture of alfalfa hay and either a control (**CON**) or an ABY-containing (**BYP**) concentrate. The CON concentrate consisted of (all values as fed) 33% corn, 20% barley, 10% wheat, 12.2% soybean meal, 8.8% palm meal, 2.5% colza meal, and 10% wheat bran. The BYP concentrate included 18% corn dried distiller grains with solubles, 18% dry citrus pulp and 8% exhausted olive cake, which replaced fully the barley and palm meal in the CON concentrate and partially replaced corn, soybean meal and wheat bran, which were reduced to 26.8%, 10.2%, and 3% in the BYP concentrate. The ABY used in this study were selected because they are produced in high amounts in the Mediterranean area and are available throughout the year. The chemical composition (DM basis) of the CON and BYP concentrates, respectively, was as follows: CP 18.1% and 19.8%, NDF 21.5% and 22.1%, ether extract (**EE**) 4.19% and 7.29%, and total starch 45.3% and 29.8%, respectively. The CP, NDF, and EE (DM basis) of the alfalfa hay were 15.2%, 51.9%, and 4.06%, respectively. The estimated ME contents, calculated according to INRA (2018) were 2.57, 2.59, and 1.94 Mcal/kg for the CON and BYP concentrates and alfalfa hay, respectively. Sheep were fed twice daily (0900 and 1800 h) at an intake level of 45 g of DM/kg of BW^0.75^ to prevent feed selection (NRC, 2007).

Each of the 2 experimental periods lasted for 6 wk, with the first 5 wk being for diet adaptation. In the last week of each period, ruminal content was collected through the cannula of each sheep at 0, 3, and 6 h after the morning feeding. In each period, sampling was conducted on 2 nonconsecutive days. Ruminal contents (approximately 100 g) were collected in sterile containers and homogenized and then approximately 60 g were freeze-dried to analyze the ruminal microbiome. The rest of the ruminal content was filtered through 4 layers of cheesecloth and the pH of the obtained fluid was immediately measured (Crisson Basic 20 pH-meter, Crisson Instruments, Barcelona, Spain). Samples of the fluid (2 mL) were mixed with 2 mL HCl (0.5 *N*) and frozen (−20°C) until analysis of VFA and ammonia concentrations. Chemical composition of alfalfa hay and concentrates was determined by AOAC International (2005) and Van Soest et al. (1991) methods as described by Marcos et al. (2020). Total starch (ID 996.11) was determined according to the AOAC procedures (AOAC International, 2005). Concentrations of VFA in ruminal fluid were determined by GC as detailed by de Evan et al. (2020) using a Shimadzu GC 2010 chromatograph (Shimadzu Europa GmbH, Duisburg, Germany). Ammonia concentrations were analyzed by the phenol-hypochlorite method with an Epoch spectrophotometer (Biotek Instruments, Swindon, UK) as described de Evan et al. (2020). Within each period, the ruminal content samples obtained in the 2 sampling days were pooled by sheep and sampling time, and were homogenized using a MM 200 mill (Retsch, GmbH, Haan, Germany). Sample processing, genomic DNA extraction, sequencing, basecalling, and bioinformatic analysis were conducted as described in Marcos et al. (2024). One sample was excluded because all of its reads were mapped as unclassified (period 1, 6-h sampling time). Reads mapped as *Annelida*, *Arthropoda*, *Bacillariophyta*, *Brachiopoda*, *Chlorophyta, Chordata*, *Cnidaria*, *Cryptophyta*, *Echinodermata*, *Mollusca*, *Nematoda*, *Phaeophyceae*, *Platyhelminthes*, *Porifera*, *Rhodophyta*, *Rotifera*, or *Streptophyta* within phylum level were filtered out, as well as those mapped as unclassified or unclassified Eukaryote. For each assessed level (genus, phylum, and superkingdom), taxonomic features (**TF**) with a relative abundance (**RA**) below 0.01% in all samples were filtered out. Taxonomical features included microorganisms that could not be identified up to the genus level, which were labeled as “unclassified” alongside the TF at the latest identified level.

The α-diversity indexes (Chao1, Shannon, and observed) of the ruminal microbiome at the genus level were calculated using the *estimate*_*richness* function from the *phyloseq* R package (McMurdie and Holmes, 2013). The data were transformed to centered log ratio (**CLR**) using the unweighted method within the *CLR* function from the *easyCoda* R package (Greenacre, 2018). Before transformation, zero count values were imputed using the geometric Bayesian multiplicative procedure via the *cmultRepl* function of the *zComposition* R package (Palarea-Albaladejo and Martín-Fernández, 2015). To evaluate the β-diversity, the Aitchison distance between samples was calculated using the CLR-transformed data. Differences in α- and β-diversity between sheep fed the mixed diets (CON vs. BYP), as well as sampling time, individual sheep, experimental period, and the interaction between diet and sampling time, were analyzed using permutational multivariate ANOVA (**PERMANOVA**). This analysis was performed using the *adonis* function from the *vegan* R package (Oksanen, 2022). A differential abundance analysis was conducted using the CLR-transformed data at genus level to determine differentially abundant (**DA**) TF between sheep fed the mixed diets and sampling times using the *Limma* R package (Ritchie et al., 2015), accounting for the effect of sheep and the period as a blocking variable utilizing the *duplicateCorrelation* function. To control for false positives the *P*-values were adjusted by the Benjamini–Hochberg method (***P*_adj_**). A |logFC| ≥ 0.5 (the logarithm of twice the abundance between groups, where **FC** is fold change) and *P*_adj_ < 0.05 were used as threshold values. Ruminal fermentation parameters of sheep fed the mixed diets were analyzed as a crossover design with time as the repeated measure, using the PROC MIXED procedure in SAS (SAS Institute, Cary, NC) as described by Hinkka and Tuominen (2025). Before statistical analysis, the data obtained in the 2 sampling days were averaged within each period, sheep, and sampling time. Effects were declared significant at *P* < 0.05.

A total of 1,101,240 out of 3,271,199 reads were mapped as unclassified. A large number of unclassified reads and TF have been previously reported in rumen samples when using Oxford Nanopore Technologies (**ONT**; Marcos et al., 2024). The final taxonomy compositions included 1,906,930, 1,926,844, and 2,169,959 reads from 23 rumen content samples, classified into 279, 39, and 4 TF at the genus, phylum, and superkingdom levels, respectively. Bacteria, mainly from the *Firmicutes*, *Bacteroidetes*, and *Proteobacteria* phyla, were the most abundant microorganisms, accounting for up to 83.3% of total reads within the final taxonomy composition. The RA of eukaryotes, archaea, and viruses were 12.8%, 3.89%, and 0.018% of total reads within the final taxonomy composition. Ruminal microbiome composition agrees with previous studies on sheep, though some differences were observed, likely because of variations in the sequencing technologies used or diet, among other factors (Belanche et al., 2019; Greenwood et al., 2022).

Differences in the Chao1 index and β-diversity at both the phylum and genus levels were observed between sheep fed the CON and BYP diets (*P* = 0.037, 0.003, and 0.002, respectively; Table 1). Variations in the composition of fermentable substrates of the 2 experimental diets may explain these differences. Belanche et al. (2012) suggested that a higher complexity of microbial populations is necessary for the efficient utilization of fibrous substrates, such as those present in the BYP concentrate. The greater EE content of the BYP concentrate could have influenced the ruminal microbiome as well. The BYP concentrate contained more polyphenols than the control concentrate (9.91 and 5.81 mg/kg, respectively; Marcos et al., 2020) as a result of inclusion of citrus pulp and olive cake, which may influence the ruminal microbiome (Scicutella et al., 2023).

**Table 1:** Variance explained (R^2^) by diet (CON vs. BYP diets), sampling time (0, 3, and 6 h after feeding), sheep, period, and the interaction between diet and sampling time, along with *P*-values from PERMANOVA for α-diversity indexes and β-diversity across taxonomical levels when comparing sheep fed different diets

Item	α-Diversity						β-Diversity					
	Chao1		Shannon		Observed		Superkingdom		Phylum		Genus	
	*P*-value	R^2^	*P*-value	R^2^	*P*-value	R^2^	*P*-value	R^2^	*P*-value	R^2^	*P*-value	R^2^
Diet	0.037	0.214	0.44	0.016	0.82	0.003	0.27	0.045	0.003	0.103	0.002	0.072
Sampling time	0.97	0.003	0.14	0.099	0.66	0.041	0.45	0.065	0.04	0.099	<0.001	0.149
Period	0.23	0.077	0.1	0.069	0.49	0.022	0.009	0.176	0.003	0.127	<0.001	0.074
Sheep	0.92	0.026	<0.001	0.516	0.15	0.29	0.18	0.167	<0.001	0.292	<0.001	0.277
Diet × sampling time	0.57	0.06	0.64	0.022	0.89	0.011	0.26	0.09	0.64	0.043	0.45	0.057
Total variance explained		0.38		0.721		0.366		0.544		0.664		0.629

No differences in α diversity indexes were observed between sampling times (*P* ≥ 0.100) but differences were observed in β-diversity at the phylum and genus levels (*P* = 0.040 and < 0.001, respectively). Variations in β-diversity across sampling times are expected because of changes in microbial abundance during the feed degradation process (Gruninger et al., 2019). Similarly, Belanche et al. (2012) reported differences in the abundance of certain microorganisms in the rumen of cattle at different postprandial times, attributing these variations to differences in microbial growth rates and feed colonization. Differences in Shannon index was observed between sheep (*P* ≤ 0.008), and the effect of sheep on β-diversity accounted for an average of 24.5% of the total variance. Host genetics have been recognized as an important factor influencing the ruminal microbiome (Weimer et al., 2010). The individual variation among sheep on α- and β-diversity was more important than the effect of the diet, likely attributable to the similar forage-to-concentrate ratio of both diets.

The abundance of the *Succinimonas* genus after 3 h of feeding was greater in sheep fed the BYP diet than in CON-fed sheep (logFC = −2.55; *P*_adj_-value = 0.021), although this genus is known as starch degrading (Belanche et al., 2019). Nevertheless, *Succinimonas* can utilize glucose (Bryant et al., 1958). Dried citrus pulp has been reported to contain a significant content of sucrose and some glucose (Pacheco et al., 2019). Sucrose can be rapidly hydrolyzed in the rumen (Weisbjerg et al., 1998) and provide further glucose that can be utilized by the genus *Succinimonas*. The genera *Bradyrhizobium*, *Klebsiella*, *Pantoea*, and *Puccinia* were overabundant at 3 h postfeeding compared with 0 h (immediately before feeding) in sheep fed either the CON or BYP diets (Table 2). Although commonly found in the rumen, *Klebsiella* is considered pathogenic bacteria in dairy ruminant farms (Zadoks et al., 2011). Other overabundant microorganisms may be plant-pathogenic or plant-associated microorganisms (Auffret et al., 2017) that are concomitantly ingested with the diet. It is probable that these microorganisms are transient and nonfunctional, which could explain why differences were only observed after 3 h of feeding. However, these microorganisms must be displaced by primary colonizing ruminal microorganisms (Gruninger et al., 2019), and thus their presence might hinder feed degradation. The abundance of *Pantoea* in sheep fed the CON diet was also greater at 6 h than at 0 h, which might suggest that it is not just a transient microorganism. Auffret et al. (2017) observed that this bacterium was more abundant in beef cattle fed mixed diets than in cattle fed fibrous diets and was highly correlated with the *Proteobacteria*/(*Firmicutes* + *Bacteroidetes*) ratio, an indicator for rumen dysbiosis, suggesting that epiphytic microorganisms ingested with the feed might indeed play a role in the rumen. An unclassified *Microbacteriaceae* from the *Micrococcales* order was overabundant after 3 h of feeding compared with 0 h in BYP-fed sheep. This microorganism is probably another plant-associated microorganism. On the contrary, an unclassified *Saccharomycetes* was overabundant in CON-fed sheep at 0 h compared with 3 h. *Saccharomyces cerevisiae* is one of the few yeasts capable of growing under anaerobiosis (Ishtar Snoek and Yde Steensma, 2007) and thus could be functional within the rumen. The higher starch content in the CON diet and its fermentation could explain the greater abundance of the amylolytic bacteria *Lactobacillus* after 3 h in CON-fed sheep compared with those fed the BYP diet, as well as the greater abundance of *Stenotrophomonas*, which has been associated with subacute ruminal acidosis in dairy cattle (Hu et al., 2022). In any case, the higher logFC observed in sheep fed the CON diet, along with the lower number of DA microorganisms in sheep fed the BYP diet, suggest that the ruminal microbiome was less resilient and more susceptible to postprandial changes with the CON diet.

**Table 2:** Differentially abundant taxonomical features at genus level between sampling times (0, 3, and 6 h after feeding) in sheep fed the CON and BYP concentrates^1^

Genera	0 vs. 3 h		0 vs. 6 h		3 vs. 6 h	
	CON	BYP	CON	BYP	CON	BYP
*Bradyrhizobium*	4.39***	2.90*			−2.94*	
*Klebsiella*	3.89***	3.16**				
*Lactobacillus*	1.50*					
*Pantoea*	2.83***	1.89*	2.29*			
*Puccinia*	4.24***	3.02*				
*Stenotrophomonas*			2.76**			
Unclassified *Microbacteriaceae*		2.12*				
Unclassified *Saccharomycetes*	−2.48*					

1 The first value indicates the logFC whereas asterisks indicate the *P*_adj_-value. Positive numbers indicate greater abundance in the latest hour and negative numbers indicate greater abundance in the earliest hour.

* *P*_adj_ < 0.05,

** *P*_adj_ < 0.01,

*** *P*_adj_ < 0.001.

Ruminal fermentation parameters before feeding (0 h) were similar to those reported by Marcos et al. (2020) in dairy goats given mixed diets including the same concentrates (CON and BYP), and the average values across sampling times were comparable to those reported by others for sheep fed mixed diets including alfalfa hay and concentrate (Ramos et al., 2009). No significant differences (Table 3) were observed between CON and BYP diets in any ruminal fermentation parameter, excepting a trend (*P* = 0.051) to lower acetate proportions with the BYP diet. These results indicate that inclusion of ABY in the concentrate did not impair the ruminal fermentation although it modulated the ruminal microbiome. However, differences between sampling times were observed in all ruminal parameters (*P* ≤ 0.020), as well as significant interactions between diet and sampling time in both ammonia and total VFA concentrations (*P* ≤ 0.042). In CON-fed sheep, concentrations of ammonia and total VFA, and propionate proportions were greatest at 3 h, whereas acetate proportions and acetate-to-propionate ratio were lowest. In contrast, no significant postprandial changes in these parameters were observed in sheep fed the BYP diet. The BYP diet consisted of a wider range of substrates with different fermentation rates resulting from the more diverse array of carbohydrates in the ABY (i.e., sugars, pectin, and other nonstarch polysaccharides), which may have promoted a more stable, complex, and active microbial consortium (Belanche et al., 2012, 2019), leading to a more sustained and efficient fermentation process that might result in better synchronization and utilization of available energy and nitrogen by ruminal microorganisms. The steady concentrations of both total VFA and ammonia over the postprandial period observed for BYP diet would support this hypothesis. The lower proportions of minor VFA (calculated as the sum of isobutyrate, valerate, and isovalerate) observed at 3 h after feeding in the BYP-fed sheep could be related to protein degradation because some minor VFA (isovalerate and isobutyrate) are produced by the microbial deamination of some AA (Stewart et al., 1997). Nonetheless, the lower proportions of minor VFA in the BYP diet might also indicate a greater uptake of these VFA by cellulolytic bacteria (Roman-Garcia et al., 2021). The pH decreased after feeding for both diets, which agrees with previous observations (Ramos et al., 2009).

**Table 3:** Ruminal fermentation parameters at different sampling times (0, 3, and 6 h after feeding) in sheep fed the CON and BYP concentrates^1^

Item	Diet	Sampling time (h)			SEM	*P-*value		
		0	3	6		Diet	Time	Diet × time
pH	CON	7.13^a^	6.19^b^	6.50^b^	0.046	0.11	<0.001	0.82
	BYP	7.03^a^	6.11^b^	6.43^b^				
NH_3_ (mg/L)	CON	85.1^a^	147^b^	79.8^a^	13.79	0.36	0.001	0.042
	BYP	99.7	98.0	56.8				
Total VFA (mmol/L)	CON	94.5^a^	172^b^	122^a^	6.8	0.36	0.008	0.040
	BYP	132	167	125				
Molar proportion (mol/100 mol)								
Acetate	CON	68.6^a^	66.6^b^	67.1^ab^	0.20	0.051	0.007	0.066
	BYP	66.2	65.3	66.0				
Propionate	CON	14.7^a^	20.2^b^	18.0^ab^	0.62	0.26	0.001	0.24
	BYP	17.1	20.3	19.2				
Butyrate	CON	11.4	9.12	10.8	0.34	0.10	0.020	0.33
	BYP	11.8	10.8	11.2				
Minor VFA^2^	CON	5.30	4.13	4.05	0.293	0.34	0.001	0.97
	BYP	4.90^a^	3.65^b^	3.63^b^				
Acetate:propionate	CON	4.69^a^	3.32^b^	3.74^b^	0.123	0.16	<0.001	0.098
	BYP	3.92	3.25	3.46				

a,b Within each parameter and diet, average values for each sampling time not sharing the same superscript differ (*P* < 0.05).

1 Effect of period was only significant for acetate proportion (*P* = 0.032). No period × time interactions (*P* ≥ 0.210) were detected by any parameter.

2 Calculated as the sum of isobutyrate, valerate, and isovalerate.

Replacing conventional feedstuffs with a mixture of agroindustrial byproducts induced changes in the ruminal microbiome of sheep. Although no significant changes in ruminal fermentation parameters were observed, the inclusion of byproducts appeared to balance ruminal fermentation, preventing large fluctuations in total VFA and ammonia concentrations in the postprandial period. This was likely because of the more diverse substrates (i.e., sugars, pectins, and other nonstarch polysaccharides) and compounds (i.e., polyphenols) present in the byproducts, their variable fermentation rates, and associated shifts in the ruminal microbiome. A more stable rumen environment could potentially enhance dairy ruminant performance. However, further studies are needed to confirm these results.
